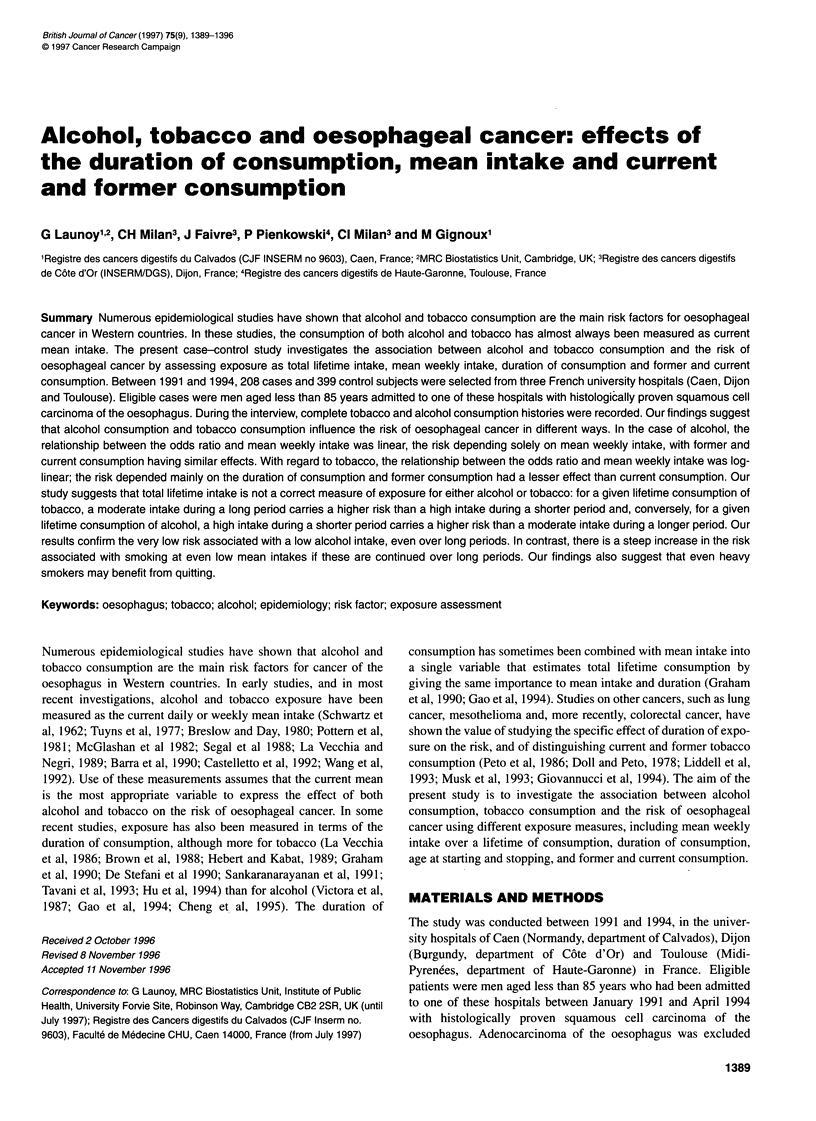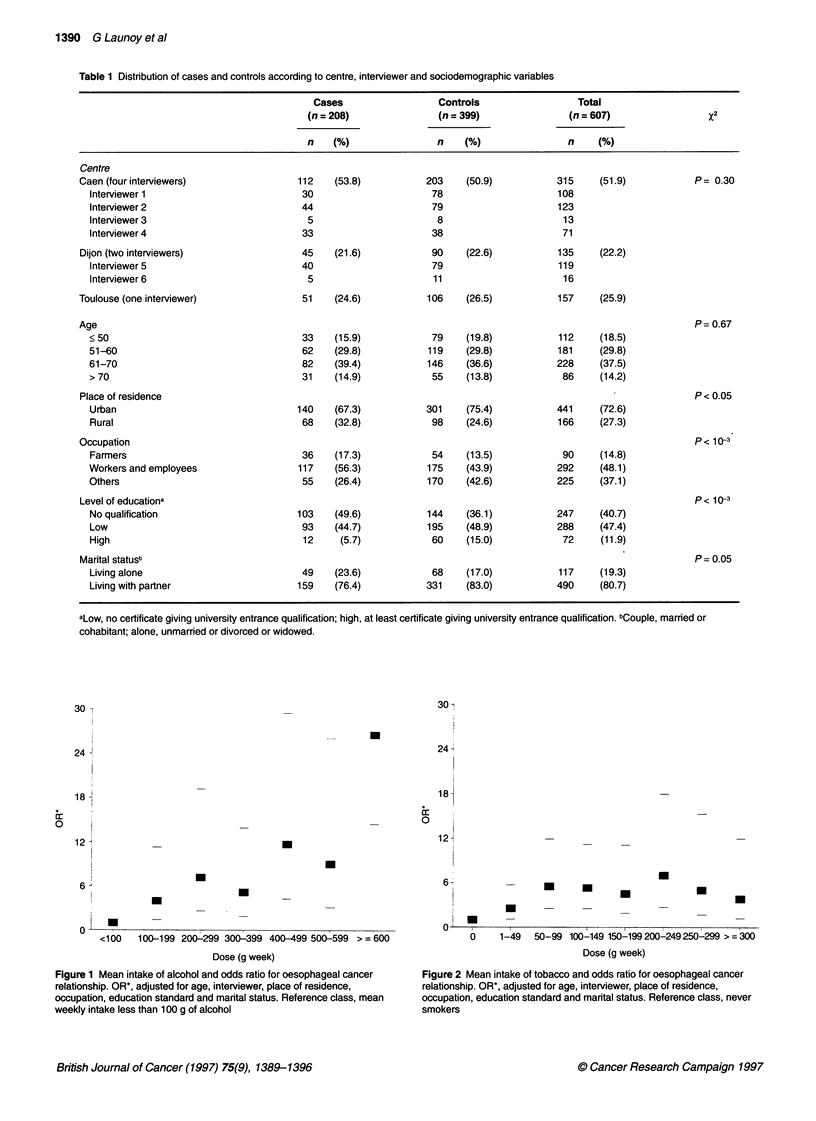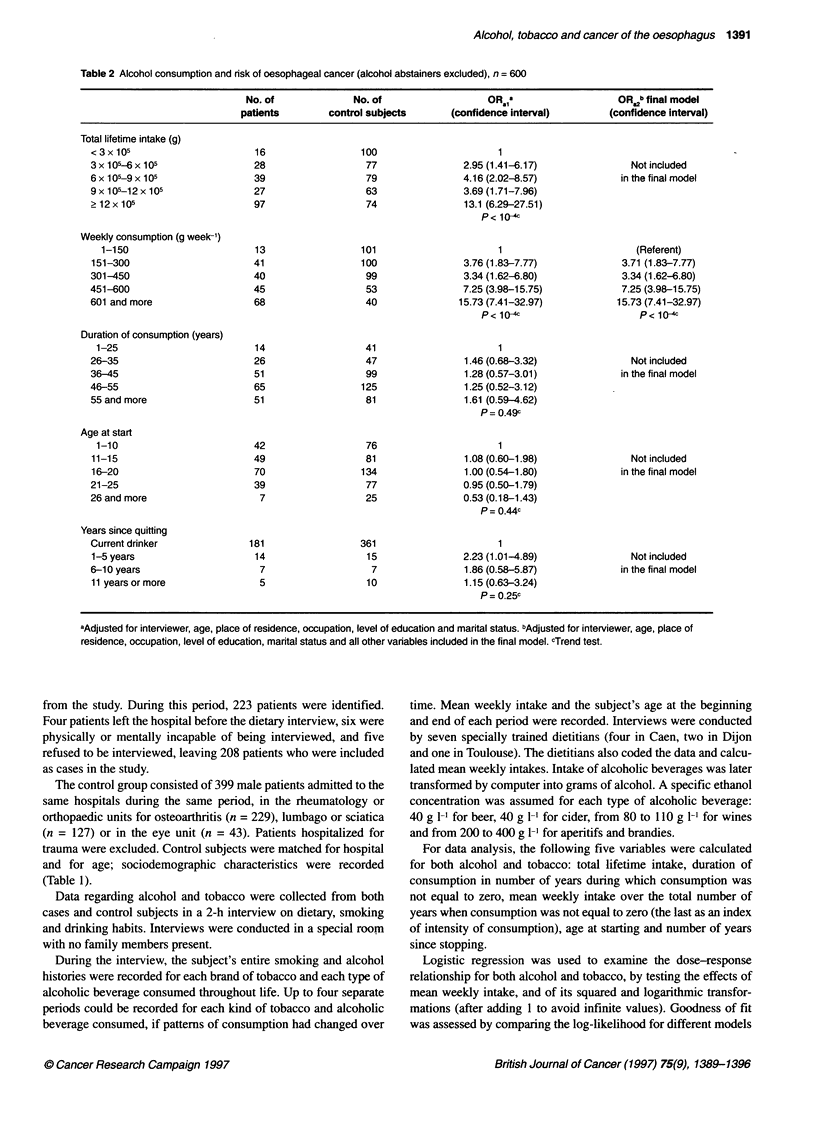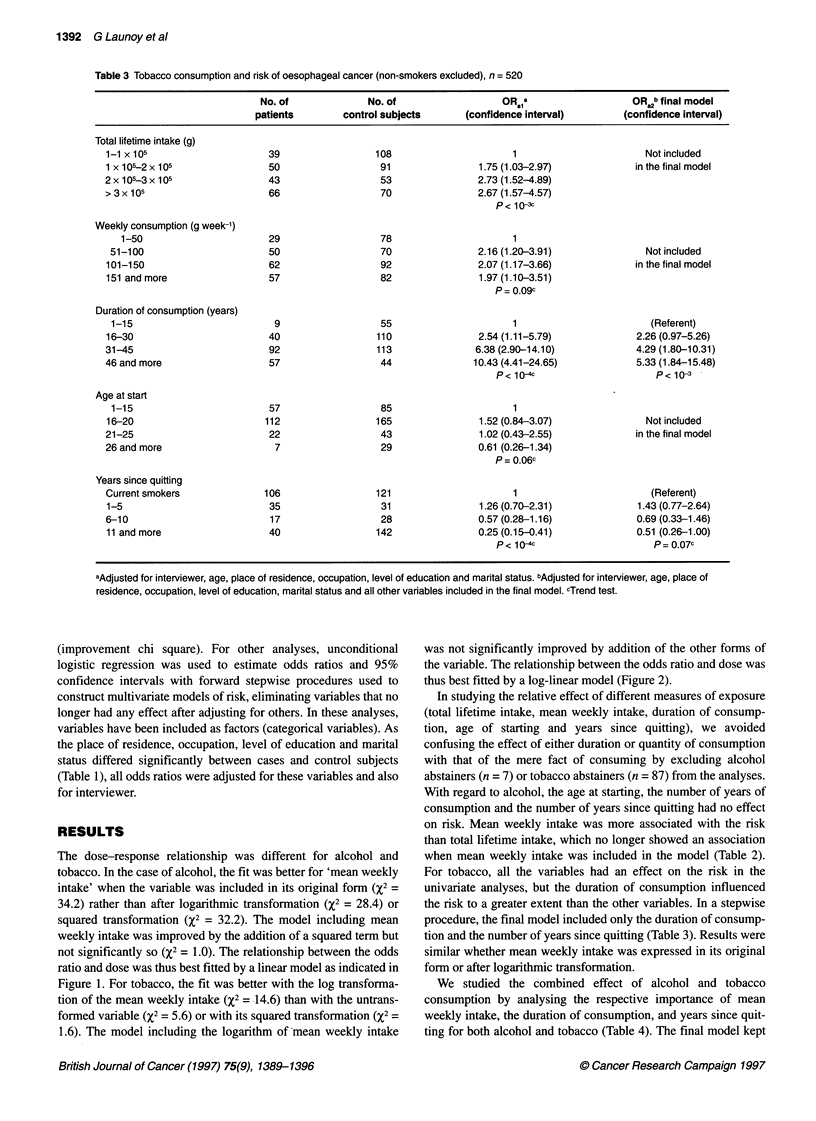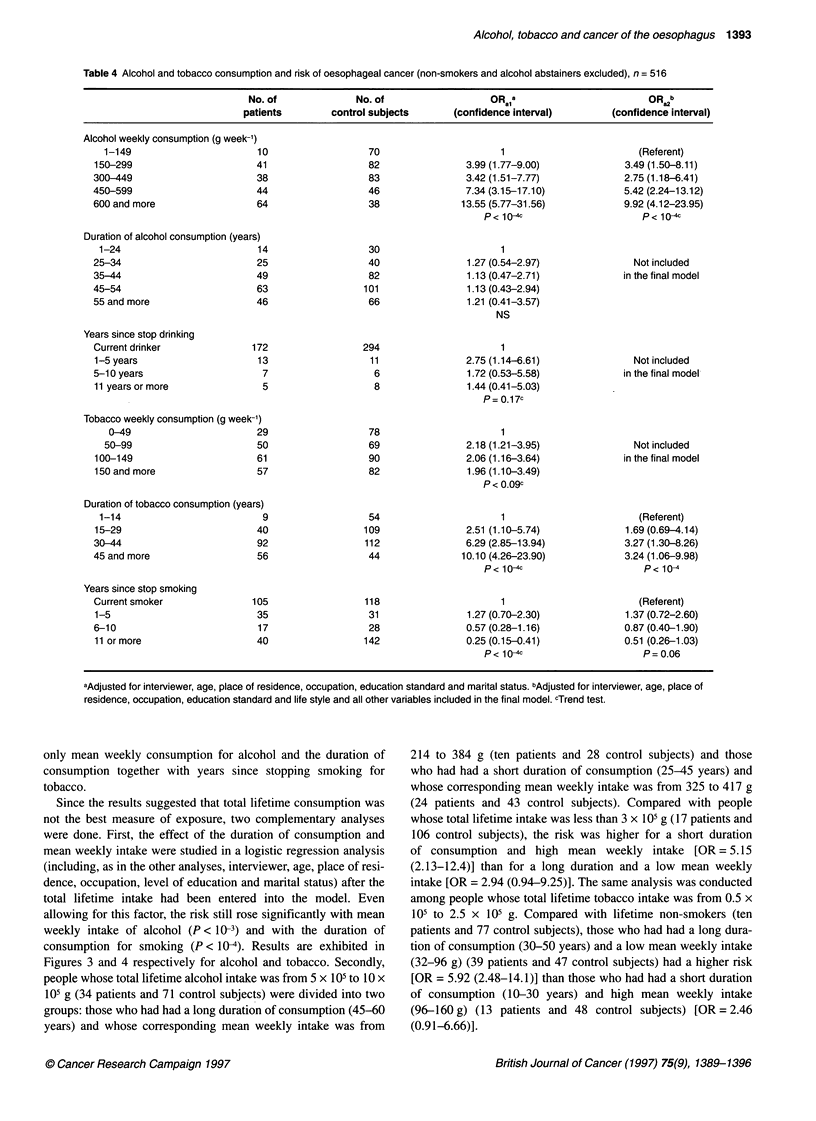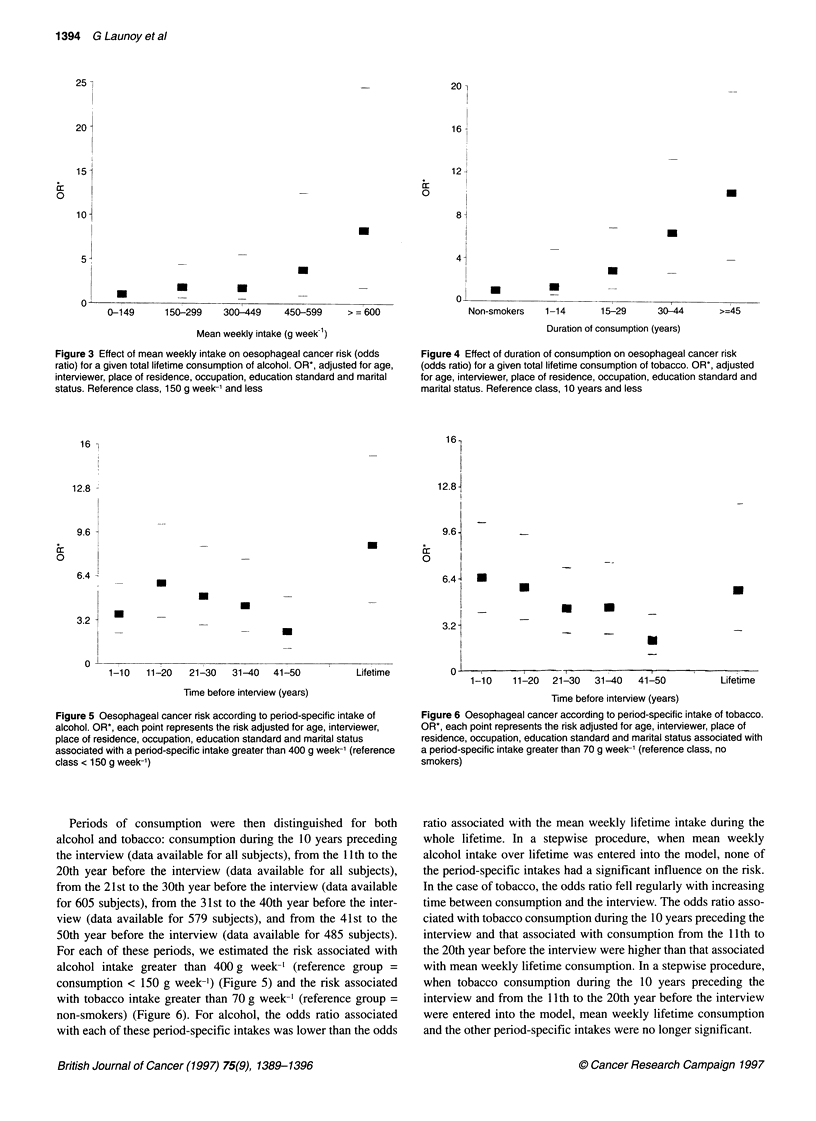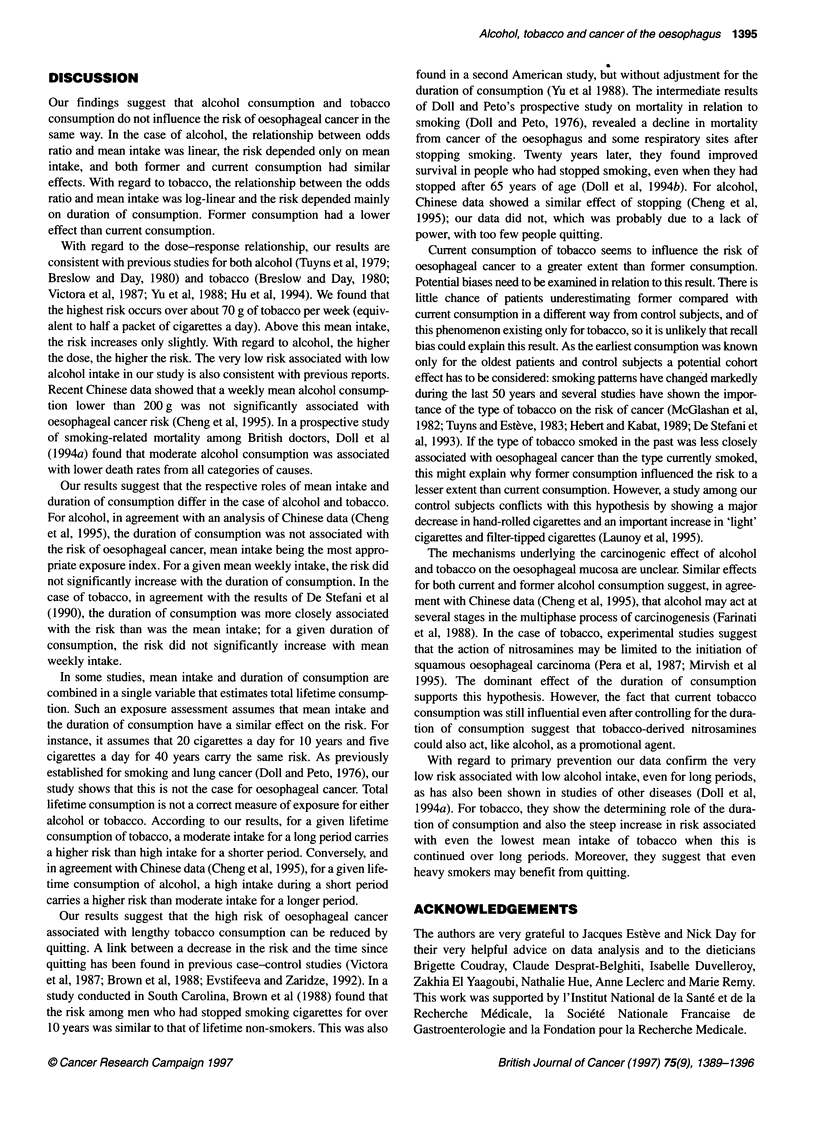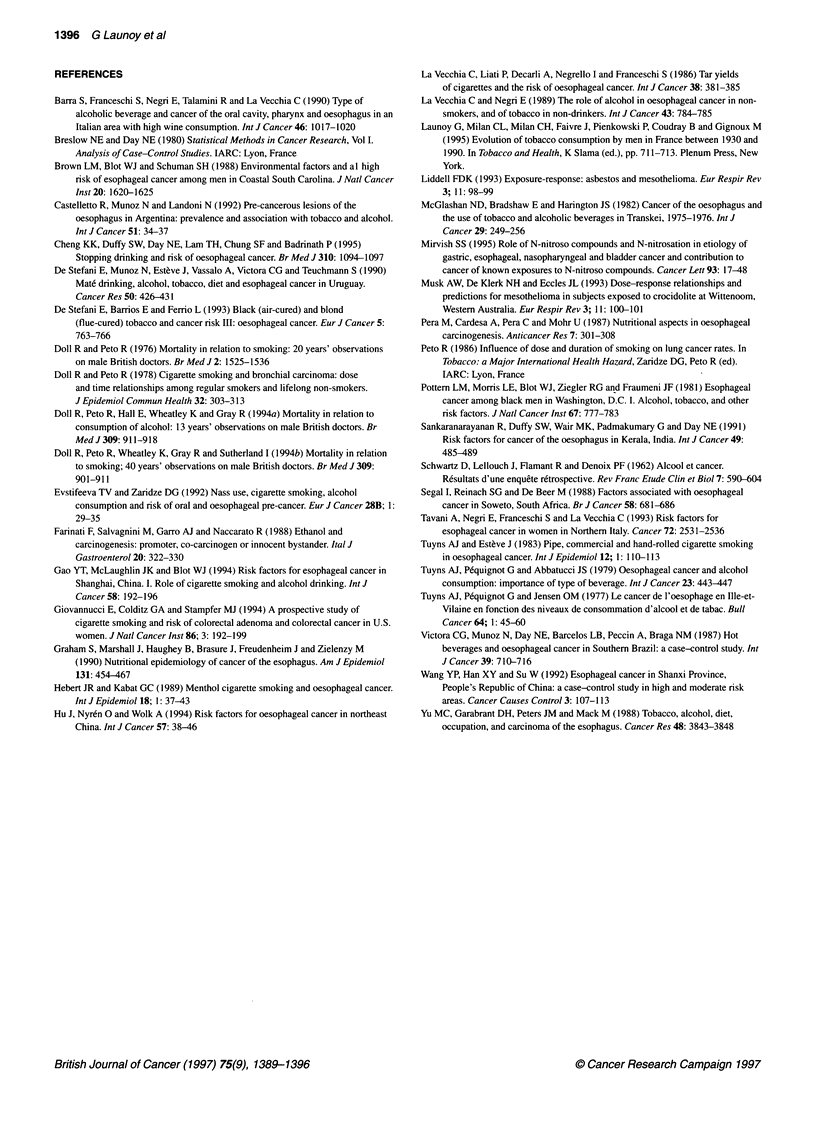# Alcohol, tobacco and oesophageal cancer: effects of the duration of consumption, mean intake and current and former consumption.

**DOI:** 10.1038/bjc.1997.236

**Published:** 1997

**Authors:** G. Launoy, C. H. Milan, J. Faivre, P. Pienkowski, C. I. Milan, M. Gignoux

**Affiliations:** Registre des cancers digestifs du Calvados (CJF INSERM no 9603), Caen, France.

## Abstract

Numerous epidemiological studies have shown that alcohol and tobacco consumption are the main risk factors for oesophageal cancer in Western countries. In these studies, the consumption of both alcohol and tobacco has almost always been measured as current mean intake. The present case-control study investigates the association between alcohol and tobacco consumption and the risk of oesophageal cancer by assessing exposure as total lifetime intake, mean weekly intake, duration of consumption and former and current consumption. Between 1991 and 1994, 208 cases and 399 control subjects were selected from three French university hospitals (Caen, Dijon and Toulouse). Eligible cases were men aged less than 85 years admitted to one of these hospitals with histologically proven squamous cell carcinoma of the oesophagus. During the interview, complete tobacco and alcohol consumption histories were recorded. Our findings suggest that alcohol consumption and tobacco consumption influence the risk of oesophageal cancer in different ways. In the case of alcohol, the relationship between the odds ratio and mean weekly intake was linear, the risk depending solely on mean weekly intake, with former and current consumption having similar effects. With regard to tobacco, the relationship between the odds ratio and mean weekly intake was log-linear; the risk depended mainly on the duration of consumption and former consumption had a lesser effect than current consumption. Our study suggests that total lifetime intake is not a correct measure of exposure for either alcohol or tobacco: for a given lifetime consumption of tobacco, a moderate intake during a long period carries a higher risk than a high intake during a shorter period and, conversely, for a given lifetime consumption of alcohol, a high intake during a shorter period carries a higher risk than a moderate intake during a longer period. Our results confirm the very low risk associated with a low alcohol intake, even over long periods. In contrast, there is a steep increase in the risk associated with smoking at even low mean intakes if these are continued over long periods. Our findings also suggest that even heavy smokers may benefit from quitting.


British Journal of Cancer (1997) 75(9), 1389–1396
© 1997 Cancer Research Campaign

### Alcohol, tobacco and oesophageal cancer: effects of the duration of consumption, mean intake and current and former consumption

G Launoy[1,2], CH Milan[3], J Faivre[3], P Pienkowski[4], Cl Milan[3] and M Gignoux[1]

[1]Registre des cancers digestifs du Calvados (CJF INSERM no 9603), Caen, France; [2]MRC Biostatistics Unit, Cambridge, UK; [3]Registre des cancers digestifs de Côte d'Or (INSERM/DGS), Dijon, France; [4]Registre des cancers digestifs de Haute-Garonne, Toulouse, France

**Summary** Numerous epidemiological studies have shown that alcohol and tobacco consumption are the main risk factors for oesophageal cancer in Western countries. In these studies, the consumption of both alcohol and tobacco has almost always been measured as current mean intake. The present case–control study investigates the association between alcohol and tobacco consumption and the risk of oesophageal cancer by assessing exposure as total lifetime intake, mean weekly intake, duration of consumption and former and current consumption. Between 1991 and 1994, 208 cases and 399 control subjects were selected from three French university hospitals (Caen, Dijon and Toulouse). Eligible cases were men aged less than 85 years admitted to one of these hospitals with histologically proven squamous cell carcinoma of the oesophagus. During the interview, complete tobacco and alcohol consumption histories were recorded. Our findings suggest that alcohol consumption and tobacco consumption influence the risk of oesophageal cancer in different ways. In the case of alcohol, the relationship between the odds ratio and mean weekly intake was linear, the risk depending solely on mean weekly intake, with former and current consumption having similar effects. With regard to tobacco, the relationship between the odds ratio and mean weekly intake was log-linear; the risk depended mainly on the duration of consumption and former consumption had a lesser effect than current consumption. Our study suggests that total lifetime intake is not a correct measure of exposure for either alcohol or tobacco: for a given lifetime consumption of tobacco, a moderate intake during a long period carries a higher risk than a high intake during a shorter period and, conversely, for a given lifetime consumption of alcohol, a high intake during a shorter period carries a higher risk than a moderate intake during a longer period. Our results confirm the very low risk associated with a low alcohol intake, even over long periods. In contrast, there is a steep increase in the risk associated with smoking at even low mean intakes if these are continued over long periods. Our findings also suggest that even heavy smokers may benefit from quitting.

**Keywords:** oesophagus; tobacco; alcohol; epidemiology; risk factor; exposure assessment

Numerous epidemiological studies have shown that alcohol and tobacco consumption are the main risk factors for cancer of the oesophagus in Western countries. In early studies, and in most recent investigations, alcohol and tobacco exposure have been measured as the current daily or weekly mean intake (Schwartz et al, 1962; Tuyns et al, 1977; Breslow and Day, 1980; Pottern et al, 1981; McGlashan et al 1982; Segal et al 1988; La Vecchia and Negri, 1989; Barra et al, 1990; Castelletto et al, 1992; Wang et al, 1992). Use of these measurements assumes that the current mean is the most appropriate variable to express the effect of both alcohol and tobacco on the risk of oesophageal cancer. In some recent studies, exposure has also been measured in terms of the duration of consumption, although more for tobacco (La Vecchia et al, 1986; Brown et al, 1988; Hebert and Kabat, 1989; Graham et al, 1990; De Stefani et al 1990; Sankaranarayanan et al, 1991; Tavani et al, 1993; Hu et al, 1994) than for alcohol (Victora et al, 1987; Gao et al, 1994; Cheng et al, 1995). The duration of consumption has sometimes been combined with mean intake into a single variable that estimates total lifetime consumption by giving the same importance to mean intake and duration (Graham et al, 1990; Gao et al, 1994). Studies on other cancers, such as lung cancer, mesothelioma and, more recently, colorectal cancer, have shown the value of studying the specific effect of duration of exposure on the risk, and of distinguishing current and former tobacco consumption (Peto et al, 1986; Doll and Peto, 1978; Liddell et al, 1993; Musk et al, 1993; Giovannucci et al, 1994). The aim of the present study is to investigate the association between alcohol consumption, tobacco consumption and the risk of oesophageal cancer using different exposure measures, including mean weekly intake over a lifetime of consumption, duration of consumption, age at starting and stopping, and former and current consumption.

*Received 2 October 1996*
*Revised 8 November 1996*
*Accepted 11 November 1996*

*Correspondence to*: G Launoy, MRC Biostatistics Unit, Institute of Public Health, University Forvie Site, Robinson Way, Cambridge CB2 2SR, UK (until July 1997); Registre des Cancers digestifs du Calvados (CJF Inserm no. 9603), Faculté de Médecine CHU, Caen 14000, France (from July 1997)

### MATERIALS AND METHODS

The study was conducted between 1991 and 1994, in the university hospitals of Caen (Normandy, department of Calvados), Dijon (Burgundy, department of Côte d'Or) and Toulouse (Midi-Pyrenées, department of Haute-Garonne) in France. Eligible patients were men aged less than 85 years who had been admitted to one of these hospitals between January 1991 and April 1994 with histologically proven squamous cell carcinoma of the oesophagus. Adenocarcinoma of the oesophagus was excluded

**Table 1** Distribution of cases and controls according to centre, interviewer and sociodemographic variables

| | Cases (*n* = 208) | | Controls (*n* = 399) | | Total (*n* = 607) | | $\chi^2$ |
|---|---|---|---|---|---|---|---|
| | *n* | (%) | *n* | (%) | *n* | (%) | |
| *Centre* | | | | | | | |
| Caen (four interviewers) | 112 | (53.8) | 203 | (50.9) | 315 | (51.9) | $P = 0.30$ |
| Interviewer 1 | 30 | | 78 | | 108 | | |
| Interviewer 2 | 44 | | 79 | | 123 | | |
| Interviewer 3 | 5 | | 8 | | 13 | | |
| Interviewer 4 | 33 | | 38 | | 71 | | |
| Dijon (two interviewers) | 45 | (21.6) | 90 | (22.6) | 135 | (22.2) | |
| Interviewer 5 | 40 | | 79 | | 119 | | |
| Interviewer 6 | 5 | | 11 | | 16 | | |
| Toulouse (one interviewer) | 51 | (24.6) | 106 | (26.5) | 157 | (25.9) | |
| Age | | | | | | | $P = 0.67$ |
| ≤ 50 | 33 | (15.9) | 79 | (19.8) | 112 | (18.5) | |
| 51–60 | 62 | (29.8) | 119 | (29.8) | 181 | (29.8) | |
| 61–70 | 82 | (39.4) | 146 | (36.6) | 228 | (37.5) | |
| > 70 | 31 | (14.9) | 55 | (13.8) | 86 | (14.2) | |
| Place of residence | | | | | | | $P < 0.05$ |
| Urban | 140 | (67.3) | 301 | (75.4) | 441 | (72.6) | |
| Rural | 68 | (32.8) | 98 | (24.6) | 166 | (27.3) | |
| Occupation | | | | | | | $P < 10^{-3}$ |
| Farmers | 36 | (17.3) | 54 | (13.5) | 90 | (14.8) | |
| Workers and employees | 117 | (56.3) | 175 | (43.9) | 292 | (48.1) | |
| Others | 55 | (26.4) | 170 | (42.6) | 225 | (37.1) | |
| Level of education[a] | | | | | | | $P < 10^{-3}$ |
| No qualification | 103 | (49.6) | 144 | (36.1) | 247 | (40.7) | |
| Low | 93 | (44.7) | 195 | (48.9) | 288 | (47.4) | |
| High | 12 | (5.7) | 60 | (15.0) | 72 | (11.9) | |
| Marital status[b] | | | | | | | $P = 0.05$ |
| Living alone | 49 | (23.6) | 68 | (17.0) | 117 | (19.3) | |
| Living with partner | 159 | (76.4) | 331 | (83.0) | 490 | (80.7) | |

[a]Low, no certificate giving university entrance qualification; high, at least certificate giving university entrance qualification. [b]Couple, married or cohabitant; alone, unmarried or divorced or widowed.

**Figure 1** Mean intake of alcohol and odds ratio for oesophageal cancer relationship. OR*, adjusted for age, interviewer, place of residence, occupation, education standard and marital status. Reference class, mean weekly intake less than 100 g of alcohol

**Figure 2** Mean intake of tobacco and odds ratio for oesophageal cancer relationship. OR*, adjusted for age, interviewer, place of residence, occupation, education standard and marital status. Reference class, never smokers

© Cancer Research Campaign 1997

**Table 2** Alcohol consumption and risk of oesophageal cancer (alcohol abstainers excluded), $n = 600$

| | No. of patients | No. of control subjects | $OR_{a1}$[a] (confidence interval) | $OR_{a2}$[b] final model (confidence interval) |
|---|---|---|---|---|
| Total lifetime intake (g) | | | | |
| $< 3 \times 10^5$ | 16 | 100 | 1 | |
| $3 \times 10^5$–$6 \times 10^5$ | 28 | 77 | 2.95 (1.41–6.17) | Not included |
| $6 \times 10^5$–$9 \times 10^5$ | 39 | 79 | 4.16 (2.02–8.57) | in the final model |
| $9 \times 10^5$–$12 \times 10^5$ | 27 | 63 | 3.69 (1.71–7.96) | |
| $\geq 12 \times 10^5$ | 97 | 74 | 13.1 (6.29–27.51) | |
| | | | $P < 10^{-4}$[c] | |
| Weekly consumption (g $week^{-1}$) | | | | |
| 1–150 | 13 | 101 | 1 | (Referent) |
| 151–300 | 41 | 100 | 3.76 (1.83–7.77) | 3.71 (1.83–7.77) |
| 301–450 | 40 | 99 | 3.34 (1.62–6.80) | 3.34 (1.62–6.80) |
| 451–600 | 45 | 53 | 7.25 (3.98–15.75) | 7.25 (3.98–15.75) |
| 601 and more | 68 | 40 | 15.73 (7.41–32.97) | 15.73 (7.41–32.97) |
| | | | $P < 10^{-4}$[c] | $P < 10^{-4}$[c] |
| Duration of consumption (years) | | | | |
| 1–25 | 14 | 41 | 1 | |
| 26–35 | 26 | 47 | 1.46 (0.68–3.32) | Not included |
| 36–45 | 51 | 99 | 1.28 (0.57–3.01) | in the final model |
| 46–55 | 65 | 125 | 1.25 (0.52–3.12) | |
| 55 and more | 51 | 81 | 1.61 (0.59–4.62) | |
| | | | $P = 0.49$[c] | |
| Age at start | | | | |
| 1–10 | 42 | 76 | 1 | |
| 11–15 | 49 | 81 | 1.08 (0.60–1.98) | Not included |
| 16–20 | 70 | 134 | 1.00 (0.54–1.80) | in the final model |
| 21–25 | 39 | 77 | 0.95 (0.50–1.79) | |
| 26 and more | 7 | 25 | 0.53 (0.18–1.43) | |
| | | | $P = 0.44$[c] | |
| Years since quitting | | | | |
| Current drinker | 181 | 361 | 1 | |
| 1–5 years | 14 | 15 | 2.23 (1.01–4.89) | Not included |
| 6–10 years | 7 | 7 | 1.86 (0.58–5.87) | in the final model |
| 11 years or more | 5 | 10 | 1.15 (0.63–3.24) | |
| | | | $P = 0.25$[c] | |

[a]Adjusted for interviewer, age, place of residence, occupation, level of education and marital status. [b]Adjusted for interviewer, age, place of residence, occupation, level of education, marital status and all other variables included in the final model. [c]Trend test.

from the study. During this period, 223 patients were identified. Four patients left the hospital before the dietary interview, six were physically or mentally incapable of being interviewed, and five refused to be interviewed, leaving 208 patients who were included as cases in the study.

The control group consisted of 399 male patients admitted to the same hospitals during the same period, in the rheumatology or orthopaedic units for osteoarthritis ($n$ = 229), lumbago or sciatica ($n$ = 127) or in the eye unit ($n$ = 43). Patients hospitalized for trauma were excluded. Control subjects were matched for hospital and for age; sociodemographic characteristics were recorded (Table 1).

Data regarding alcohol and tobacco were collected from both cases and control subjects in a 2-h interview on dietary, smoking and drinking habits. Interviews were conducted in a special room with no family members present.

During the interview, the subject's entire smoking and alcohol histories were recorded for each brand of tobacco and each type of alcoholic beverage consumed throughout life. Up to four separate periods could be recorded for each kind of tobacco and alcoholic beverage consumed, if patterns of consumption had changed over time. Mean weekly intake and the subject's age at the beginning and end of each period were recorded. Interviews were conducted by seven specially trained dietitians (four in Caen, two in Dijon and one in Toulouse). The dietitians also coded the data and calculated mean weekly intakes. Intake of alcoholic beverages was later transformed by computer into grams of alcohol. A specific ethanol concentration was assumed for each type of alcoholic beverage: 40 g $l^{-1}$ for beer, 40 g $l^{-1}$ for cider, from 80 to 110 g $l^{-1}$ for wines and from 200 to 400 g $l^{-1}$ for aperitifs and brandies.

For data analysis, the following five variables were calculated for both alcohol and tobacco: total lifetime intake, duration of consumption in number of years during which consumption was not equal to zero, mean weekly intake over the total number of years when consumption was not equal to zero (the last as an index of intensity of consumption), age at starting and number of years since stopping.

Logistic regression was used to examine the dose–response relationship for both alcohol and tobacco, by testing the effects of mean weekly intake, and of its squared and logarithmic transformations (after adding 1 to avoid infinite values). Goodness of fit was assessed by comparing the log-likelihood for different models

© Cancer Research Campaign 1997 

**Table 3** Tobacco consumption and risk of oesophageal cancer (non-smokers excluded), $n = 520$

| | No. of patients | No. of control subjects | $OR_{a1}$[a] (confidence interval) | $OR_{a2}$[b] final model (confidence interval) |
|---|---|---|---|---|
| Total lifetime intake (g) | | | | |
| $1–1 \times 10^5$ | 39 | 108 | 1 | Not included |
| $1 \times 10^5–2 \times 10^5$ | 50 | 91 | 1.75 (1.03–2.97) | in the final model |
| $2 \times 10^5–3 \times 10^5$ | 43 | 53 | 2.73 (1.52–4.89) | |
| $> 3 \times 10^5$ | 66 | 70 | 2.67 (1.57–4.57) | |
| | | | $P < 10^{-3}$[c] | |
| Weekly consumption (g week$^{-1}$) | | | | |
| 1–50 | 29 | 78 | 1 | |
| 51–100 | 50 | 70 | 2.16 (1.20–3.91) | Not included |
| 101–150 | 62 | 92 | 2.07 (1.17–3.66) | in the final model |
| 151 and more | 57 | 82 | 1.97 (1.10–3.51) | |
| | | | $P = 0.09$[c] | |
| Duration of consumption (years) | | | | |
| 1–15 | 9 | 55 | 1 | (Referent) |
| 16–30 | 40 | 110 | 2.54 (1.11–5.79) | 2.26 (0.97–5.26) |
| 31–45 | 92 | 113 | 6.38 (2.90–14.10) | 4.29 (1.80–10.31) |
| 46 and more | 57 | 44 | 10.43 (4.41–24.65) | 5.33 (1.84–15.48) |
| | | | $P < 10^{-4}$[c] | $P < 10^{-3}$ |
| Age at start | | | | |
| 1–15 | 57 | 85 | 1 | |
| 16–20 | 112 | 165 | 1.52 (0.84–3.07) | Not included |
| 21–25 | 22 | 43 | 1.02 (0.43–2.55) | in the final model |
| 26 and more | 7 | 29 | 0.61 (0.26–1.34) | |
| | | | $P = 0.06$[c] | |
| Years since quitting | | | | |
| Current smokers | 106 | 121 | 1 | (Referent) |
| 1–5 | 35 | 31 | 1.26 (0.70–2.31) | 1.43 (0.77–2.64) |
| 6–10 | 17 | 28 | 0.57 (0.28–1.16) | 0.69 (0.33–1.46) |
| 11 and more | 40 | 142 | 0.25 (0.15–0.41) | 0.51 (0.26–1.00) |
| | | | $P < 10^{-4}$[c] | $P = 0.07$[c] |

[a]Adjusted for interviewer, age, place of residence, occupation, level of education and marital status. [b]Adjusted for interviewer, age, place of residence, occupation, level of education, marital status and all other variables included in the final model. [c]Trend test.

(improvement chi square). For other analyses, unconditional logistic regression was used to estimate odds ratios and 95% confidence intervals with forward stepwise procedures used to construct multivariate models of risk, eliminating variables that no longer had any effect after adjusting for others. In these analyses, variables have been included as factors (categorical variables). As the place of residence, occupation, level of education and marital status differed significantly between cases and control subjects (Table 1), all odds ratios were adjusted for these variables and also for interviewer.

### RESULTS

The dose–response relationship was different for alcohol and tobacco. In the case of alcohol, the fit was better for 'mean weekly intake' when the variable was included in its original form ($\chi^2$ = 34.2) rather than after logarithmic transformation ($\chi^2$ = 28.4) or squared transformation ($\chi^2$ = 32.2). The model including mean weekly intake was improved by the addition of a squared term but not significantly so ($\chi^2$ = 1.0). The relationship between the odds ratio and dose was thus best fitted by a linear model as indicated in Figure 1. For tobacco, the fit was better with the log transformation of the mean weekly intake ($\chi^2$ = 14.6) than with the untransformed variable ($\chi^2$ = 5.6) or with its squared transformation ($\chi^2$ = 1.6). The model including the logarithm of mean weekly intake was not significantly improved by addition of the other forms of the variable. The relationship between the odds ratio and dose was thus best fitted by a log-linear model (Figure 2).

In studying the relative effect of different measures of exposure (total lifetime intake, mean weekly intake, duration of consumption, age of starting and years since quitting), we avoided confusing the effect of either duration or quantity of consumption with that of the mere fact of consuming by excluding alcohol abstainers ($n$ = 7) or tobacco abstainers ($n$ = 87) from the analyses. With regard to alcohol, the age at starting, the number of years of consumption and the number of years since quitting had no effect on risk. Mean weekly intake was more associated with the risk than total lifetime intake, which no longer showed an association when mean weekly intake was included in the model (Table 2). For tobacco, all the variables had an effect on the risk in the univariate analyses, but the duration of consumption influenced the risk to a greater extent than the other variables. In a stepwise procedure, the final model included only the duration of consumption and the number of years since quitting (Table 3). Results were similar whether mean weekly intake was expressed in its original form or after logarithmic transformation.

We studied the combined effect of alcohol and tobacco consumption by analysing the respective importance of mean weekly intake, the duration of consumption, and years since quitting for both alcohol and tobacco (Table 4). The final model kept

 © Cancer Research Campaign 1997

**Table 4** Alcohol and tobacco consumption and risk of oesophageal cancer (non-smokers and alcohol abstainers excluded), $n = 516$

| | No. of patients | No. of control subjects | $OR_{a1}$[a] (confidence interval) | $OR_{a2}$[b] (confidence interval) |
|---|---|---|---|---|
| Alcohol weekly consumption (g week$^{-1}$) | | | | |
| 1–149 | 10 | 70 | 1 | (Referent) |
| 150–299 | 41 | 82 | 3.99 (1.77–9.00) | 3.49 (1.50–8.11) |
| 300–449 | 38 | 83 | 3.42 (1.51–7.77) | 2.75 (1.18–6.41) |
| 450–599 | 44 | 46 | 7.34 (3.15–17.10) | 5.42 (2.24–13.12) |
| 600 and more | 64 | 38 | 13.55 (5.77–31.56) | 9.92 (4.12–23.95) |
| | | | $P < 10^{-4}$[c] | $P < 10^{-4}$[c] |
| Duration of alcohol consumption (years) | | | | |
| 1–24 | 14 | 30 | 1 | |
| 25–34 | 25 | 40 | 1.27 (0.54–2.97) | Not included |
| 35–44 | 49 | 82 | 1.13 (0.47–2.71) | in the final model |
| 45–54 | 63 | 101 | 1.13 (0.43–2.94) | |
| 55 and more | 46 | 66 | 1.21 (0.41–3.57) | |
| | | | NS | |
| Years since stop drinking | | | | |
| Current drinker | 172 | 294 | 1 | |
| 1–5 years | 13 | 11 | 2.75 (1.14–6.61) | Not included |
| 5–10 years | 7 | 6 | 1.72 (0.53–5.58) | in the final model |
| 11 years or more | 5 | 8 | 1.44 (0.41–5.03) | |
| | | | $P = 0.17$[c] | |
| Tobacco weekly consumption (g week$^{-1}$) | | | | |
| 0–49 | 29 | 78 | 1 | |
| 50–99 | 50 | 69 | 2.18 (1.21–3.95) | Not included |
| 100–149 | 61 | 90 | 2.06 (1.16–3.64) | in the final model |
| 150 and more | 57 | 82 | 1.96 (1.10–3.49) | |
| | | | $P < 0.09$[c] | |
| Duration of tobacco consumption (years) | | | | |
| 1–14 | 9 | 54 | 1 | (Referent) |
| 15–29 | 40 | 109 | 2.51 (1.10–5.74) | 1.69 (0.69–4.14) |
| 30–44 | 92 | 112 | 6.29 (2.85–13.94) | 3.27 (1.30–8.26) |
| 45 and more | 56 | 44 | 10.10 (4.26–23.90) | 3.24 (1.06–9.98) |
| | | | $P < 10^{-4}$[c] | $P < 10^{-4}$ |
| Years since stop smoking | | | | |
| Current smoker | 105 | 118 | 1 | (Referent) |
| 1–5 | 35 | 31 | 1.27 (0.70–2.30) | 1.37 (0.72–2.60) |
| 6–10 | 17 | 28 | 0.57 (0.28–1.16) | 0.87 (0.40–1.90) |
| 11 or more | 40 | 142 | 0.25 (0.15–0.41) | 0.51 (0.26–1.03) |
| | | | $P < 10^{-4}$[c] | $P = 0.06$ |

[a]Adjusted for interviewer, age, place of residence, occupation, education standard and marital status. [b]Adjusted for interviewer, age, place of residence, occupation, education standard and life style and all other variables included in the final model. [c]Trend test.

only mean weekly consumption for alcohol and the duration of consumption together with years since stopping smoking for tobacco.

Since the results suggested that total lifetime consumption was not the best measure of exposure, two complementary analyses were done. First, the effect of the duration of consumption and mean weekly intake were studied in a logistic regression analysis (including, as in the other analyses, interviewer, age, place of residence, occupation, level of education and marital status) after the total lifetime intake had been entered into the model. Even allowing for this factor, the risk still rose significantly with mean weekly intake of alcohol ($P < 10^{-3}$) and with the duration of consumption for smoking ($P < 10^{-4}$). Results are exhibited in Figures 3 and 4 respectively for alcohol and tobacco. Secondly, people whose total lifetime alcohol intake was from $5 \times 10^5$ to $10 \times 10^5$ g (34 patients and 71 control subjects) were divided into two groups: those who had had a long duration of consumption (45–60 years) and whose corresponding mean weekly intake was from 214 to 384 g (ten patients and 28 control subjects) and those who had had a short duration of consumption (25–45 years) and whose corresponding mean weekly intake was from 325 to 417 g (24 patients and 43 control subjects). Compared with people whose total lifetime intake was less than $3 \times 10^5$ g (17 patients and 106 control subjects), the risk was higher for a short duration of consumption and high mean weekly intake [OR = 5.15 (2.13–12.4)] than for a long duration and a low mean weekly intake [OR = 2.94 (0.94–9.25)]. The same analysis was conducted among people whose total lifetime tobacco intake was from $0.5 \times 10^5$ to $2.5 \times 10^5$ g. Compared with lifetime non-smokers (ten patients and 77 control subjects), those who had had a long duration of consumption (30–50 years) and a low mean weekly intake (32–96 g) (39 patients and 47 control subjects) had a higher risk [OR = 5.92 (2.48–14.1)] than those who had had a short duration of consumption (10–30 years) and high mean weekly intake (96–160 g) (13 patients and 48 control subjects) [OR = 2.46 (0.91–6.66)].

© Cancer Research Campaign 1997

**Figure 3** Effect of mean weekly intake on oesophageal cancer risk (odds ratio) for a given total lifetime consumption of alcohol. OR*, adjusted for age, interviewer, place of residence, occupation, education standard and marital status. Reference class, 150 g week$^{-1}$ and less

**Figure 4** Effect of duration of consumption on oesophageal cancer risk (odds ratio) for a given total lifetime consumption of tobacco. OR*, adjusted for age, interviewer, place of residence, occupation, education standard and marital status. Reference class, 10 years and less

**Figure 5** Oesophageal cancer risk according to period-specific intake of alcohol. OR*, each point represents the risk adjusted for age, interviewer, place of residence, occupation, education standard and marital status associated with a period-specific intake greater than 400 g week$^{-1}$ (reference class < 150 g week$^{-1}$)

**Figure 6** Oesophageal cancer according to period-specific intake of tobacco. OR*, each point represents the risk adjusted for age, interviewer, place of residence, occupation, education standard and marital status associated with a period-specific intake greater than 70 g week$^{-1}$ (reference class, no smokers)

Periods of consumption were then distinguished for both alcohol and tobacco: consumption during the 10 years preceding the interview (data available for all subjects), from the 11th to the 20th year before the interview (data available for all subjects), from the 21st to the 30th year before the interview (data available for 605 subjects), from the 31st to the 40th year before the interview (data available for 579 subjects), and from the 41st to the 50th year before the interview (data available for 485 subjects). For each of these periods, we estimated the risk associated with alcohol intake greater than 400 g week$^{-1}$ (reference group = consumption < 150 g week$^{-1}$) (Figure 5) and the risk associated with tobacco intake greater than 70 g week$^{-1}$ (reference group = non-smokers) (Figure 6). For alcohol, the odds ratio associated with each of these period-specific intakes was lower than the odds ratio associated with the mean weekly lifetime intake during the whole lifetime. In a stepwise procedure, when mean weekly alcohol intake over lifetime was entered into the model, none of the period-specific intakes had a significant influence on the risk. In the case of tobacco, the odds ratio fell regularly with increasing time between consumption and the interview. The odds ratio associated with tobacco consumption during the 10 years preceding the interview and that associated with consumption from the 11th to the 20th year before the interview were higher than that associated with mean weekly lifetime consumption. In a stepwise procedure, when tobacco consumption during the 10 years preceding the interview and from the 11th to the 20th year before the interview were entered into the model, mean weekly lifetime consumption and the other period-specific intakes were no longer significant.

© Cancer Research Campaign 1997

### DISCUSSION

Our findings suggest that alcohol consumption and tobacco consumption do not influence the risk of oesophageal cancer in the same way. In the case of alcohol, the relationship between odds ratio and mean intake was linear, the risk depended only on mean intake, and both former and current consumption had similar effects. With regard to tobacco, the relationship between the odds ratio and mean intake was log-linear and the risk depended mainly on duration of consumption. Former consumption had a lower effect than current consumption.

With regard to the dose–response relationship, our results are consistent with previous studies for both alcohol (Tuyns et al, 1979; Breslow and Day, 1980) and tobacco (Breslow and Day, 1980; Victora et al, 1987; Yu et al, 1988; Hu et al, 1994). We found that the highest risk occurs over about 70 g of tobacco per week (equivalent to half a packet of cigarettes a day). Above this mean intake, the risk increases only slightly. With regard to alcohol, the higher the dose, the higher the risk. The very low risk associated with low alcohol intake in our study is also consistent with previous reports. Recent Chinese data showed that a weekly mean alcohol consumption lower than 200 g was not significantly associated with oesophageal cancer risk (Cheng et al, 1995). In a prospective study of smoking-related mortality among British doctors, Doll et al (1994*a*) found that moderate alcohol consumption was associated with lower death rates from all categories of causes.

Our results suggest that the respective roles of mean intake and duration of consumption differ in the case of alcohol and tobacco. For alcohol, in agreement with an analysis of Chinese data (Cheng et al, 1995), the duration of consumption was not associated with the risk of oesophageal cancer, mean intake being the most appropriate exposure index. For a given mean weekly intake, the risk did not significantly increase with the duration of consumption. In the case of tobacco, in agreement with the results of De Stefani et al (1990), the duration of consumption was more closely associated with the risk than was the mean intake; for a given duration of consumption, the risk did not significantly increase with mean weekly intake.

In some studies, mean intake and duration of consumption are combined in a single variable that estimates total lifetime consumption. Such an exposure assessment assumes that mean intake and the duration of consumption have a similar effect on the risk. For instance, it assumes that 20 cigarettes a day for 10 years and five cigarettes a day for 40 years carry the same risk. As previously established for smoking and lung cancer (Doll and Peto, 1976), our study shows that this is not the case for oesophageal cancer. Total lifetime consumption is not a correct measure of exposure for either alcohol or tobacco. According to our results, for a given lifetime consumption of tobacco, a moderate intake for a long period carries a higher risk than high intake for a shorter period. Conversely, and in agreement with Chinese data (Cheng et al, 1995), for a given lifetime consumption of alcohol, a high intake during a short period carries a higher risk than moderate intake for a longer period.

Our results suggest that the high risk of oesophageal cancer associated with lengthy tobacco consumption can be reduced by quitting. A link between a decrease in the risk and the time since quitting has been found in previous case–control studies (Victora et al, 1987; Brown et al, 1988; Evstifeeva and Zaridze, 1992). In a study conducted in South Carolina, Brown et al (1988) found that the risk among men who had stopped smoking cigarettes for over 10 years was similar to that of lifetime non-smokers. This was also found in a second American study, but without adjustment for the duration of consumption (Yu et al 1988). The intermediate results of Doll and Peto's prospective study on mortality in relation to smoking (Doll and Peto, 1976), revealed a decline in mortality from cancer of the oesophagus and some respiratory sites after stopping smoking. Twenty years later, they found improved survival in people who had stopped smoking, even when they had stopped after 65 years of age (Doll et al, 1994*b*). For alcohol, Chinese data showed a similar effect of stopping (Cheng et al, 1995); our data did not, which was probably due to a lack of power, with too few people quitting.

Current consumption of tobacco seems to influence the risk of oesophageal cancer to a greater extent than former consumption. Potential biases need to be examined in relation to this result. There is little chance of patients underestimating former compared with current consumption in a different way from control subjects, and of this phenomenon existing only for tobacco, so it is unlikely that recall bias could explain this result. As the earliest consumption was known only for the oldest patients and control subjects a potential cohort effect has to be considered: smoking patterns have changed markedly during the last 50 years and several studies have shown the importance of the type of tobacco on the risk of cancer (McGlashan et al, 1982; Tuyns and Estève, 1983; Hebert and Kabat, 1989; De Stefani et al, 1993). If the type of tobacco smoked in the past was less closely associated with oesophageal cancer than the type currently smoked, this might explain why former consumption influenced the risk to a lesser extent than current consumption. However, a study among our control subjects conflicts with this hypothesis by showing a major decrease in hand-rolled cigarettes and an important increase in 'light' cigarettes and filter-tipped cigarettes (Launoy et al, 1995).

The mechanisms underlying the carcinogenic effect of alcohol and tobacco on the oesophageal mucosa are unclear. Similar effects for both current and former alcohol consumption suggest, in agreement with Chinese data (Cheng et al, 1995), that alcohol may act at several stages in the multiphase process of carcinogenesis (Farinati et al, 1988). In the case of tobacco, experimental studies suggest that the action of nitrosamines may be limited to the initiation of squamous oesophageal carcinoma (Pera et al, 1987; Mirvish et al 1995). The dominant effect of the duration of consumption supports this hypothesis. However, the fact that current tobacco consumption was still influential even after controlling for the duration of consumption suggest that tobacco-derived nitrosamines could also act, like alcohol, as a promotional agent.

With regard to primary prevention our data confirm the very low risk associated with low alcohol intake, even for long periods, as has also been shown in studies of other diseases (Doll et al, 1994*a*). For tobacco, they show the determining role of the duration of consumption and also the steep increase in risk associated with even the lowest mean intake of tobacco when this is continued over long periods. Moreover, they suggest that even heavy smokers may benefit from quitting.

### ACKNOWLEDGEMENTS

The authors are very grateful to Jacques Estève and Nick Day for their very helpful advice on data analysis and to the dieticians Brigette Coudray, Claude Desprat-Belghiti, Isabelle Duvelleroy, Zakhia El Yaagoubi, Nathalie Hue, Anne Leclerc and Marie Remy. This work was supported by l'Institut National de la Santé et de la Recherche Médicale, la Société Nationale Francaise de Gastroenterologie and la Fondation pour la Recherche Medicale.

© Cancer Research Campaign 1997

### REFERENCES

Barra S, Franceschi S, Negri E, Talamini R and La Vecchia C (1990) Type of alcoholic beverage and cancer of the oral cavity, pharynx and oesophagus in an Italian area with high wine consumption. *Int J Cancer* **46**: 1017–1020

Breslow NE and Day NE (1980) *Statistical Methods in Cancer Research*, Vol I. *Analysis of Case–Control Studies*. IARC: Lyon, France

Brown LM, Blot WJ and Schuman SH (1988) Environmental factors and al high risk of esophageal cancer among men in Coastal South Carolina. *J Natl Cancer Inst* **20**: 1620–1625

Castelletto R, Munoz N and Landoni N (1992) Pre-cancerous lesions of the oesophagus in Argentina: prevalence and association with tobacco and alcohol. *Int J Cancer* **51**: 34–37

Cheng KK, Duffy SW, Day NE, Lam TH, Chung SF and Badrinath P (1995) Stopping drinking and risk of oesophageal cancer. *Br Med J* **310**: 1094–1097

De Stefani E, Munoz N, Estève J, Vassalo A, Victora CG and Teuchmann S (1990) Maté drinking, alcohol, tobacco, diet and esophageal cancer in Uruguay. *Cancer Res* **50**: 426–431

De Stefani E, Barrios E and Ferrio L (1993) Black (air-cured) and blond (flue-cured) tobacco and cancer risk III: oesophageal cancer. *Eur J Cancer* **5**: 763–766

Doll R and Peto R (1976) Mortality in relation to smoking: 20 years' observations on male British doctors. *Br Med J* **2**: 1525–1536

Doll R and Peto R (1978) Cigarette smoking and bronchial carcinoma: dose and time relationships among regular smokers and lifelong non-smokers. *J Epidemiol Commun Health* **32**: 303–313

Doll R, Peto R, Hall E, Wheatley K and Gray R (1994*a*) Mortality in relation to consumption of alcohol: 13 years' observations on male British doctors. *Br Med J* **309**: 911–918

Doll R, Peto R, Wheatley K, Gray R and Sutherland I (1994*b*) Mortality in relation to smoking; 40 years' observations on male British doctors. *Br Med J* **309**: 901–911

Evstifeeva TV and Zaridze DG (1992) Nass use, cigarette smoking, alcohol consumption and risk of oral and oesophageal pre-cancer. *Eur J Cancer* **28B**; 1: 29–35

Farinati F, Salvagnini M, Garro AJ and Naccarato R (1988) Ethanol and carcinogenesis: promoter, co-carcinogen or innocent bystander. *Ital J Gastroenterol* **20**: 322–330

Gao YT, McLaughlin JK and Blot WJ (1994) Risk factors for esophageal cancer in Shanghai, China. I. Role of cigarette smoking and alcohol drinking. *Int J Cancer* **58**: 192–196

Giovannucci E, Colditz GA and Stampfer MJ (1994) A prospective study of cigarette smoking and risk of colorectal adenoma and colorectal cancer in U.S. women. *J Natl Cancer Inst* **86**; 3: 192–199

Graham S, Marshall J, Haughey B, Brasure J, Freudenheim J and Zielenzy M (1990) Nutritional epidemiology of cancer of the esophagus. *Am J Epidemiol* **131**: 454–467

Hebert JR and Kabat GC (1989) Menthol cigarette smoking and oesophageal cancer. *Int J Epidemiol* **18**; 1: 37–43

Hu J, Nyrén O and Wolk A (1994) Risk factors for oesophageal cancer in northeast China. *Int J Cancer* **57**: 38–46

La Vecchia C, Liati P, Decarli A, Negrello I and Franceschi S (1986) Tar yields of cigarettes and the risk of oesophageal cancer. *Int J Cancer* **38**: 381–385

La Vecchia C and Negri E (1989) The role of alcohol in oesophageal cancer in non-smokers, and of tobacco in non-drinkers. *Int J Cancer* **43**: 784–785

Launoy G, Milan CL, Milan CH, Faivre J, Pienkowski P, Coudray B and Gignoux M (1995) Evolution of tobacco consumption by men in France between 1930 and 1990. In *Tobacco and Health*, K Slama (ed.), pp. 711–713. Plenum Press, New York.

Liddell FDK (1993) Exposure-response: asbestos and mesothelioma. *Eur Respir Rev* **3**; 11: 98–99

McGlashan ND, Bradshaw E and Harington JS (1982) Cancer of the oesophagus and the use of tobacco and alcoholic beverages in Transkei, 1975–1976. *Int J Cancer* **29**: 249–256

Mirvish SS (1995) Role of N-nitroso compounds and N-nitrosation in etiology of gastric, esophageal, nasopharyngeal and bladder cancer and contribution to cancer of known exposures to N-nitroso compounds. *Cancer Lett* **93**: 17–48

Musk AW, De Klerk NH and Eccles JL (1993) Dose–response relationships and predictions for mesothelioma in subjects exposed to crocidolite at Wittenoom, Western Australia. *Eur Respir Rev* **3**; 11: 100–101

Pera M, Cardesa A, Pera C and Mohr U (1987) Nutritional aspects in oesophageal carcinogenesis. *Anticancer Res* **7**: 301–308

Peto R (1986) Influence of dose and duration of smoking on lung cancer rates. In *Tobacco: a Major International Health Hazard*, Zaridze DG, Peto R (ed). IARC: Lyon, France

Pottern LM, Morris LE, Blot WJ, Ziegler RG and Fraumeni JF (1981) Esophageal cancer among black men in Washington, D.C. I. Alcohol, tobacco, and other risk factors. *J Natl Cancer Inst* **67**: 777–783

Sankaranarayanan R, Duffy SW, Wair MK, Padmakumary G and Day NE (1991) Risk factors for cancer of the oesophagus in Kerala, India. *Int J Cancer* **49**: 485–489

Schwartz D, Lellouch J, Flamant R and Denoix PF (1962) Alcool et cancer. Résultats d'une enquête rétrospective. *Rev Franc Etude Clin et Biol* **7**: 590–604

Segal I, Reinach SG and De Beer M (1988) Factors associated with oesophageal cancer in Soweto, South Africa. *Br J Cancer* **58**: 681–686

Tavani A, Negri E, Franceschi S and La Vecchia C (1993) Risk factors for esophageal cancer in women in Northern Italy. *Cancer* **72**: 2531–2536

Tuyns AJ and Estève J (1983) Pipe, commercial and hand-rolled cigarette smoking in oesophageal cancer. *Int J Epidemiol* **12**; 1: 110–113

Tuyns AJ, Péquignot G and Abbatucci JS (1979) Oesophageal cancer and alcohol consumption: importance of type of beverage. *Int J Cancer* **23**: 443–447

Tuyns AJ, Péquignot G and Jensen OM (1977) Le cancer de l'oesophage en Ille-et-Vilaine en fonction des niveaux de consommation d'alcool et de tabac. *Bull Cancer* **64**; 1: 45–60

Victora CG, Munoz N, Day NE, Barcelos LB, Peccin A, Braga NM (1987) Hot beverages and oesophageal cancer in Southern Brazil: a case–control study. *Int J Cancer* **39**: 710–716

Wang YP, Han XY and Su W (1992) Esophageal cancer in Shanxi Province, People's Republic of China: a case–control study in high and moderate risk areas. *Cancer Causes Control* **3**: 107–113

Yu MC, Garabrant DH, Peters JM and Mack M (1988) Tobacco, alcohol, diet, occupation, and carcinoma of the esophagus. *Cancer Res* **48**: 3843–3848

© Cancer Research Campaign 1997